# Structural and Enhanced Optical Properties of Stabilized γ‒Bi_2_O_3_ Nanoparticles: Effect of Oxygen Ion Vacancies

**DOI:** 10.3390/nano10061023

**Published:** 2020-05-27

**Authors:** Ashish Chhaganlal Gandhi, Chia-Liang Cheng, Sheng Yun Wu

**Affiliations:** Department of Physics, National Dong Hwa University, Hualien 97401, Taiwan; acg.gandhi@gmail.com (A.C.G.); clcheng@gms.ndhu.edu.tw (C.-L.C.)

**Keywords:** γ–Bi_2_O_3_, nanoparticles, metastable, Raman scattering, x-ray diffraction, oxygen vacancies

## Abstract

We report the synthesis of room temperature (RT) stabilized γ–Bi_2_O_3_ nanoparticles (NPs) at the expense of metallic Bi NPs through annealing in an ambient atmosphere. RT stability of the metastable γ–Bi_2_O_3_ NPs is confirmed using synchrotron radiation powder X-ray diffraction and Raman spectroscopy. γ–Bi_2_O_3_ NPs exhibited a strong red-band emission peaking at ~701 nm, covering 81% integrated intensity of photoluminescence spectra. Our findings suggest that the RT stabilization and enhanced red-band emission of γ‒Bi_2_O_3_ is mediated by excess oxygen ion vacancies generated at the octahedral O(2) sites during the annealing process.

## 1. Introduction

In recent years, body-centered-cubic (BCC) γ–Bi_2_O_3_ nanostructures have received enormous attention because of their enhanced photocatalytic performance for water purification and water splitting [1,2,3,4,5,6,7,8,9,10]. The high-temperature metastable γ–Bi_2_O_3_ is one out of nine polymorphs of Bi_2_O_3_ (α–, β–, γ–, δ–, ω–, ε–, η–, ζ– and R–phases) [11]. Schumb et al. [12] was the first to report on the preparation of γ–Bi_2_O_3_ by heating β–Bi_2_O_3_ at 750–800 °C followed by cooling in the air at 639 °C [13]. On further cooling, phase transformation occurs from γ–Bi_2_O_3_ to α–Bi_2_O_3_ in the temperature range of 368–639 °C [14]. According to Radaev et al. [15], the tetrahedral sites in the BCC structure of γ–Bi_2_O_3_ are populated by Bi^3+^ ions with a probability of 80%, suggesting 20% intrinsic vacancies [16] (given as ⎕) of both Bi and O ions. Therefore, the expected chemical formula of γ–Bi_2_O_3_ is written as ${\mathrm{Bi}}_{12}{\mathrm{Bi}}_{0.80}O_{19.20}⇋{\mathrm{Bi}}_{12}{\left[{\left({\mathrm{Bi}}_{\mathrm{Bi}}^{x}O_{3}E\right)}_{0.8}\left({⎕}_{\mathrm{Bi}}^{⁗}O_{4}\right)\right]}_{0.2}O_{16}$ according to Kröger–Vink notation [17]. E represents a 6s^2^ lone pair of electrons giving a $\left({\mathrm{Bi}}_{\mathrm{Bi}}^{x}O_{3}E\right)$ tetrahedron occupying a 2*a* symmetric site. The tetrahedrally coordinated vacancy $\left({⎕}_{\mathrm{Bi}}^{⁗}O_{4}\right)$ is statically distributed over the Bi–O lattice [15]. γ–Bi_2_O_3_ belongs to the sillenite family and is isomorphous to Bi_12_GeO_20_ (space group I23), where Bi and Ge atoms occupy 24*f* and 2*a* symmetric sites [15]. Room temperature (RT) stabilized γ–Bi_2_O_3_ was obtained through doping a foreign element at the 2*a* symmetric site [16,18,19,20,21,22,23]. However, the dopant ions could hamper the intrinsic properties of γ–Bi_2_O_3_. Hereof, in recent decades, various physical and chemical methods have been introduced for the synthesis of RT stabilized γ–Bi_2_O_3_ nanostructures with various morphologies [1,9]. Versatile forms of nanostructures could be obtained either by heating the mixture at low temperature (40–90 °C) [2,3,24,25,26,27,28,29] or in some methods at high temperature (300–800 °C) [4,6,7,30,31,32].

The synthesis of RT stabilized γ–Bi_2_O_3_ through a chemical method is usually sensitive to the preparation conditions such as reaction temperature, time, and additive types [4,20,25]. Li et al. introduced various surfactants for RT stabilization of γ–Bi_2_O_3_ [2]. Egorysheva et al. utilized ethylene glycol (EG) and polyethylene glycol [3], and Wang et al. utilized EG for the nucleation of γ–Bi_2_O_3,_ which otherwise leads to the formation of α–Bi_2_O_3_ [24]. Wang et al. reported the formation of surfactant stabilized γ–Bi_2_O_3_ due to oxygen vacancy (V_O_) defects [26]. Ahila et al. reported that the nucleation and grain growth process induced phase transformation from β– to γ–Bi_2_O_3_ by consuming surrounding β–Bi_2_O_3_ only by annealing anodic bismuth trioxide between 500 and 600 °C [32]. Liu et al. reported the synthesis of RT stabilized γ–Bi_2_O_3_ (using the solution crystallization method) over a wide temperature range (300 to 700 °C) [4]. Recently, Bandyopadhyay et al. reported the transformation from α–Bi_2_O_3_ to RT stabilized γ–Bi_2_O_3_ by the mechanical alloying method [33]. However, as discussed above, the critical factor responsible for RT stabilization of high-temperature metastable γ–Bi_2_O_3_ is still unknown. In this regard, a question remains unanswered: what could be the mechanism behind RT stabilization of γ–Bi_2_O_3_ at the nanoscale? Is it due to the finite size effect? Or is there any role of defects (such as oxygen vacancies, V_O_)?

To answer these questions, in this work, we have introduced a simple two-step physical method for the synthesis of RT stabilized γ–Bi_2_O_3_ NPs solely at the expense of Bi NPs. The proposed method uses a single parameter, annealing temperature, to prepare γ–Bi_2_O_3_ NPs from pure Bi NPs. The method is simple, cost-effective, and free of any additive such as surfactants and template agents, other metal oxides, and post-transition ions. In combination with several sensitive probes, such as synchrotron radiation powder X-ray diffractometer (PXRD), Raman and photoluminescence (PL) spectroscopy, we present a correlation for the V_O_ and RT stabilization of γ–Bi_2_O_3_ NPs. Raman spectroscopy, which is a simple and non-destructive technique, can act as a fingerprint of the various polymorphs of Bi_2_O_3_ [34]. It is also utilized in analyzing point defects such as cation and anion vacancies [35]. Similarly, PL spectroscopies can be used as a powerful tool in identifying defects, particularly for V_O_ in metal oxides [26,36]. Our experimental results indicate the formation of vacancies at B(1) and O(2) sites during annealing, of which the former could be intrinsic and later play a decisive role in the RT stabilization of γ–Bi_2_O_3_. The effect of vacancies leads to Raman peak broadening and red-shift, resulting in an inhomogeneous distorted Bi–O lattice. In particular, it is suggested that the intense red-band emission in γ–Bi_2_O_3_ is associated with V_O_ formed at O(2) sites during the annealing process. The new finding in this study is valuable in terms of providing a fundamental understanding of the RT stabilization of γ–Bi_2_O_3_ and, from an industrial point of view, creating ease in its mass production for its future use as a photocatalyst.

## 2. Materials and Methods

Annealing of Bi NPs in the air leads to the formation of bismuth oxides, which can be described as $\mathrm{Bi}\left(\mathrm{NPs}\right)+O_{2}\left(\mathrm{air}\right)\to {\mathrm{Bi}}_{2}O_{3}\left(\mathrm{NPs}\right)$ [37,38]. This route is very simple, cost-effective, and also free from any additional chemicals such as surfactants and template agents, which could introduce impurities as a byproduct [24,33]. Therefore, in this study, the synthesis of γ‒Bi_2_O_3_ NPs was carried out in two steps. In the first step, black colored Bi NPs were obtained using the physical vapor deposition (PVD) method, the detail of which has been given in our previous work [39]. In the second step, the as-obtained Bi NPs were annealed at 550 °C with a heating rate of 10 °C min^−1^ for a duration of 2 h in the air, and subsequently allowed to cool down to ambient temperature. The annealing process resulted in the formation of a whitish-yellow colored powder sample.

## 3. Results

### 3.1. Morphological and Elemental Analysis

**Figure 1:**
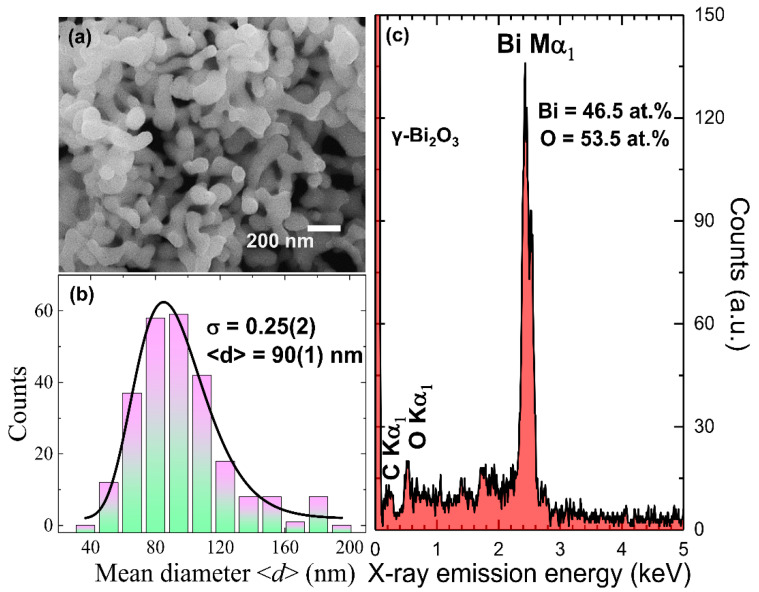
Plots of (**a**) SEM image; (**b**) the histogram of mean diameter distribution where the solid line represents long-normal distribution fit; and (**c**) EDS spectra of γ–Bi_2_O_3_ nanoparticles (NPs).

The surface morphological analysis of each sample was performed using field-emission scanning electron microscopy (FE-SEM) (JEOL JSM-6500 microscope, JEOL Ltd., Tokyo, Japan). To estimate the average atomic percentage (at. %) of the constituent elements, energy dispersive spectroscopy (EDS) (Inca x-sight model 7557, Oxford Instruments, Abingdon, UK) was utilized. For SEM and EDS analyses, the powder sample was initially dispersed in ethanol and sonicated for 30 min. Then, a drop of dispersed powder was put on the silicon wafer, and ethanol was allowed to evaporate under an infra-red lamp. The silicon wafer, along with the sample, was then mounted on a Cu-grid using carbon tape. Figure 1a shows an SEM image of well-connected γ–Bi_2_O_3_ NPs. The melting temperature of the bulk metallic Bi is 271 °C, and due to the size effect, it could reduce further to a lower value [37]. Hence, the annealing effect at such a high temperature (i.e., 550 °C) may have led to aggregation because of the melting and coalescence with neighboring particles. A histogram plot of the mean diameter <*d*> (shortest side of the particles) obtained from the SEM images is shown in Figure 1b. The solid line represents fit using a log-normal distribution function with <*d*> = 90(1) nm and the standard distribution *σ* = 0.25(2) in the particle diameter. Figure 1c shows the EDS spectra of γ–Bi_2_O_3_ NPs assigned to Bi Mα_1_ and O Kα_1_ without any impurity. The small peak of C Kα_1_ originates from the sample exposed to air. The average Bi and O at. % obtained from nine EDS spectra (Figure S1 and Table S1) for γ–Bi_2_O_3_ NPs is 46.50% and 53.50%. The obtained higher Bi/O at. % ratio ~0.87 as compared to 2/3 suggested possible oxygen deficiency in γ–Bi_2_O_3_ NPs induced during the growth process.

### 3.2. Structural Properties

**Figure 2:**
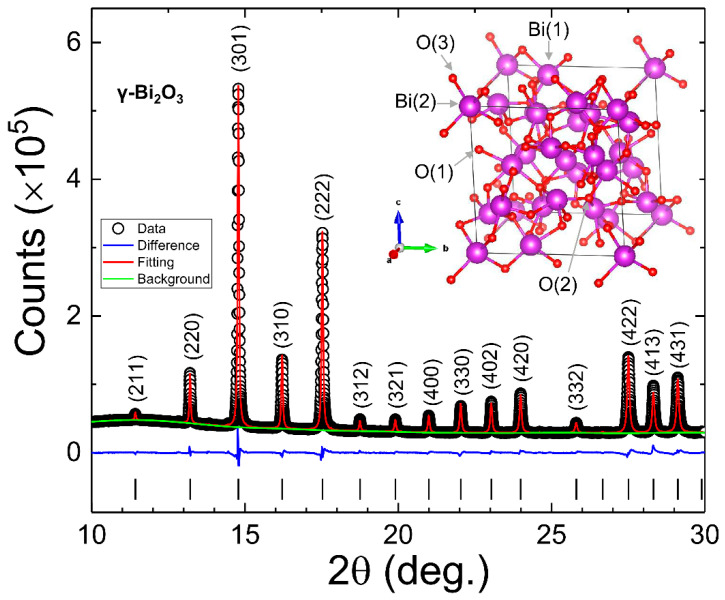
Rietveld refined (red line) PXRD spectra (open dots) of γ–Bi_2_O_3_ NPs. Green and blue lines represent the background and difference between observed and fitted PXRD spectra. A unit cell of γ–Bi_2_O_3_ is shown in the inset.

Bi_2_O_3_ with its nine polymorphs is a very complex system. Hence, there is a strong possibility that the annealing of Bi in the air could result in a mixed phase compound (i.e., a compound with two or more polymorphs of Bi_2_O_3_) [34]. Hence, synchrotron radiation PXRD was employed at the National Synchrotron Radiation Research Center in Hsinchu, Taiwan (beamline BL01C2, *λ* = 0.7749 Å) for the detailed structural characterization. Figure 2 shows the synchrotron radiation PXRD spectra (open dots), suggesting a good crystallinity of γ–Bi_2_O_3_ NPs. The most intense (301) diffraction peak at 2*θ* = 4.787° can be fitted with a Lorentzian distribution function, giving a full-width at half-maximum (FWHM) *β* = 0.04996(41)°. Using Scherrer’s formula d=kλ/βcosθ, the calculated grain size of γ–Bi_2_O_3_ is $d_{\left(301\right)}=90\;\mathrm{nm},$ which matches very well with the estimated mean diameter from the SEM images.

The structural parameters, including bismuth and oxygen site occupancies, were determined by performing Rietveld refinement of XRPD spectra using the general structure analysis system (GSAS)-II software package [40]. The sites Bi(1), Bi(2), O(1), O(2) and O(3) in the γ–Bi_2_O_3_ unit cell are demonstrated in the inset of Figure 2. The best Rietveld refined fit to the diffraction pattern (open dots) is represented by the red line in Figure 2, and the corresponding refinement parameters are given in Table 1. The fitted value of lattice constant *a* = 10.1115(1) Å (space group *I*23, No. 197) with a unit cell volume V = 1033.82(3) Å^3^ is in good agreement with the literature, showing 4% contraction in a unit cell volume with respect to the bulk [15]. Recently, *a* = 10.3106(3) Å was reported for ~24 nm γ–Bi_2_O_3_ NPs prepared using the mechanical alloying method, suggesting that the sample preparation method plays a significant role in defining the size and the structural properties [33]. The fitted value of occupancy at the Bi(1), O(1), and O(3) sites remains very close to 1, whereas the occupancies at the Bi(2) and O(2) sites are 0.943(8) and 0.861(29), respectively. The observed vacancy of Bi (V_Bi_) at Bi(2), i.e., 2*a* sites, could be intrinsic [15,16]. The excess V_O_ at O(2), i.e., 8c sites, may have been induced during the growth process of γ–Bi_2_O_3_. The structural results suggest that the proposed annealing treatment to obtain RT stabilized pure γ–Bi_2_O_3_ NPs without any impurity phase or polymorphs of Bi_2_O_3_ from metallic Bi NPs is very mild, which eases its mass preparation.

### 3.3. Raman Spectroscopy

The PXRD measurements above are mainly dominated by the heavier Bi atoms. Due to this, small displacement in the relatively lighter oxygen yields negligible deviation in the diffraction spectra. Furthermore, the Bi(1) and O(1) sites have lower symmetry, and the displacement does not result in a lower symmetry site. On the other hand, optical Raman spectroscopy is sensitive to the Bi–O bonding and the PL spectroscopy to various defects. Hence, red-shift in frequencies and an increase in the line-width of phonon modes can give evidence about the inhomogeneous distribution. To study the phonon vibration, a micro Raman spectrometer (Renishaw, UK with 1800 lines/mm grating) coupled with a microscope (Leica, Wetzlar, Germany) was utilized. A 532 nm wavelength laser with 1% power was used as the excitation. The exposer time was 60 s. A QE65000 charge-coupled device imaging spectrometer was used to detect the PL spectra of the sample. A Q-switched diode-pumped solid-state laser (266 nm) acted as the pumping light source.

**Figure 3:**
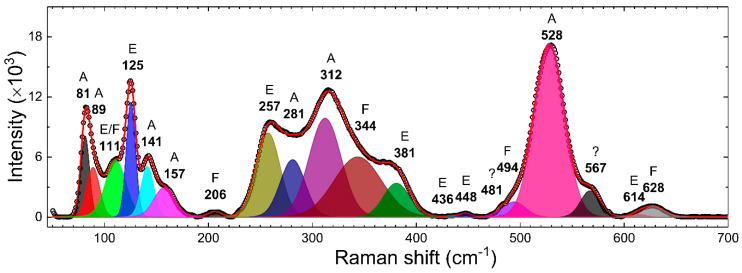
Deconvoluted (red line) room temperature (RT) Raman spectra (open dots) of γ–Bi_2_O_3_ NPs. The fitted values of frequencies and the corresponding vibration modes (A, E, and F) are shown.

RT Raman spectra of γ–Bi_2_O_3_ NPs were recorded from 50 to 1100 cm^−1^. Figure 3 shows the deconvoluted (red line) Raman spectra (open dots) of γ–Bi_2_O_3_ NPs over 50 to 700 cm^−1^. The fitted value of frequencies (FWHM) and integrated area (I.A.) are summarized in Table 2. The BCC γ–Bi_2_O_3_ belongs to the sillenite family, and on the basis of one formula unit per primitive cell, a group-theoretical analysis predicts 40 zone-center optical phonon modes: Γ = 8A (totally symmetric) + 8E (doubly degenerate) + 25F (triply degenerate) [41]. Except for one F mode, which is acoustic, all modes are Raman-active, and the F modes are infrared-active. The Raman spectra of γ–Bi_2_O_3_ NPs displayed 20 vibration modes, consistent with the literature, where so far, only 8 to 15 modes have been resolved in the same range [4,30,34,42]. The Raman bands in Figure 3 were assigned by comparing with the reported values for γ–Bi_2_O_3_ and the theoretical calculations in the literature [30,41,42,43,44,45]. A very weak mode at 481 cm^−1^ and a broad mode at 567 cm^−1^, which have been reported for γ–Bi_2_O_3_, are so far not assigned to any vibrational mode. The Raman modes below ~650 cm^−1^ are usually assigned to the internal bismuth–oxygen framework and are indicative of several breathing, stretching, rocking, and bending modes of Bi–O polyhedra in γ–Bi_2_O_3_ [30,41,42,43,44,45]. Raman modes in 70–190 cm^−1^ are quite sharp, whereas most of the modes from 190 to 660 cm^−1^ are quite broadened with FWHM varying between 14 and 54 cm^−1^. Salazar-Pérez et al. [30] reported the Raman spectra from oxygen-deficient (confirmed using EDS) γ–Bi_2_O_3_ NPs prepared by annealing 10 nm Bi NPs in the air at 700–750 °C for a duration of 30 min. Comparatively, (i) in this work γ–Bi_2_O_3_ is formed at a relatively low temperature, which could be possibly related to the use of different size Bi NPs; (ii) all the modes from 190 to 660 cm^−1^ show red-shift with a magnitude varying between 6 and 25 cm^−^^1^; (iii) the intensity of modes at 89, 206, and 281 cm^−1^ is relatively low, and to the naked eye, it appears that these peaks are broadened. The broadening and red-shift give evidence about the inhomogeneous distorted Bi–O lattice. The inhomogeneity could be due to the size effect or formed defects such as Bi and O vacancies, as observed from PXRD.

### 3.4. Photoluminescence Spectroscopy

**Figure 4:**
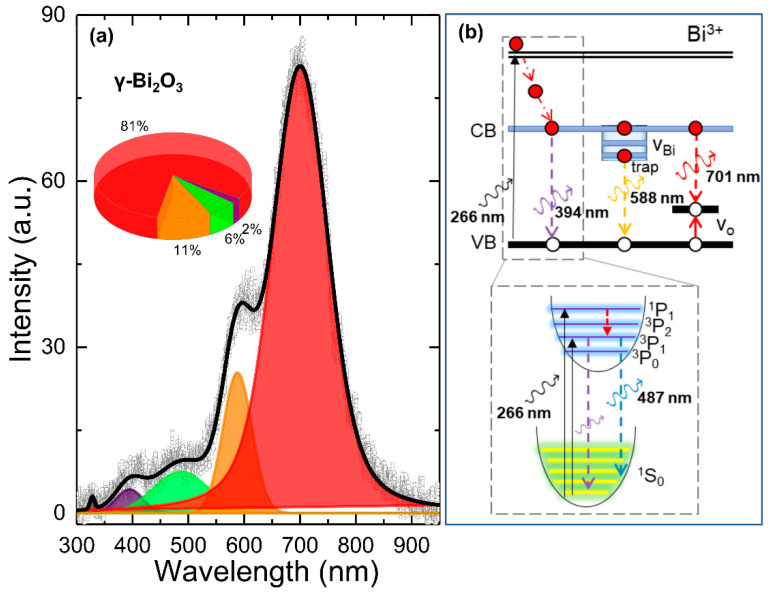
(**a**) Deconvoluted (black line) RT PL spectra (open dots) of γ–Bi_2_O_3_ NPs. Inset in (**a**) shows a pie chart representing the integrated area of deconvoluted peaks; (**b**) a schematic energy level diagram for Bi^3+^ valence state.

PL spectroscopy is a powerful tool and has been used in examining electronic transitions and information in the search for impurities, defects, and optical bandgap in semiconductor materials. PL emission from different Bi_2_O_3_ polymorphs is mainly attributed to Bi^3+^ and Bi^2+^ intrinsic transitions and complex defects such as V_O_. The luminescence from Bi^3+^ appears from the blue region to the green region under UV excitation, attributed either to ^3^P_1_ → ^1^S_0_ transition or charge-transfer transitions between oxygen ligands and Bi^3+^ ions [46,47]. The emission from Bi^2+^ is attributed to ^2^P_3/2_(1) → ^2^P_1/2_ transitions, giving rise to luminescence spectra that peak in the wavelength range 591–637 nm under UV excitation [48,49]. Recently, the low-energy red-band emission has been attributed to crystal defects or defect levels associated with V_O_ or bismuth interstitials formed during the growth process [50,51]. Figure 4a presents the RT PL spectra (open dots) of γ–Bi_2_O_3_ showing a strong and broad emission from ~350 to 900 nm centered at around 700 nm. The deconvolution of the PL spectra (black line) was carried out using the sum of four emission bands centered at 394, 487, 588, and 701 nm (Table 3), which agrees with the reported spectra in [26]. A schematic energy level diagram for the Bi^3+^ valence state is shown in Figure 4b. Bi^3+^ ions have a 6s^2^ configuration with ground state ^1^S_0_. The excited sp state gives a triplet (^3^P_0_, ^3^P_1_, ^3^P_2_) for parallel spin and a single ^1^P_1_ for antiparallel spin. The excitation usually occurs from the ^1^S_0_ ground state to the ^3^P_1_ and ^1^P_1_ excited states [49]. The estimated value of a direct bandgap for γ–Bi_2_O_3_ NPs using a UV-visible diffuse reflectance spectrum is ~2.9 eV (434 nm) (data are not shown), which is slightly lower than the reported value of 2.95 eV [3] and higher than 2.76–2.83 eV [30]. Therefore, the emission peak at 394 nm (2% of PL spectra) can be indexed as the band-to-band recombination in a direct transition manner (^3^P_1_ → ^1^S_0_). The emission peak at 487 nm (about 6% of PL spectra) should be attributed to blue-green emission corresponding to Bi^3+^ ions (bottom panel in Figure 4b). The yellow-orange emission at 588 nm (about 11% of PL spectra) is from an impurity trap associated with the surface V_O_ interacting with interfacial bismuth vacancies (V_Bi_) [24]. In general, high PL intensity indicates a higher recombination rate of the photo-excited electron-hole pair and vice versa. The strongest low-energy red emission band (81% of PL spectra), located around 701 nm, could be associated with various structural defects such as V_O_, V_Bi_, and an interstitial defect that may have formed during the growth process. The effect of annealing at such a high temperature (550 °C) is more favorable to generate vacancies rather than interstitial defects if the energy and chemical balance between the NPs and the ambient gas is taken into consideration. Therefore, both V_O_ and V_Bi_ could have been formed simultaneously. However, the observed oxygen deficiency from EDS, vacancies at O(2) sites from PXRD, a red-shift, and a line-width broadening from Raman spectra suggest that V_O_ density defect levels could lead to intense red-band emission. Wang et al. also reported similar red-band emission centered at 705 nm and attributed to a high density of V_O_ on the surface of γ–Bi_2_O_3_ [26]. Kumari et al. also reported emission maxima between 660 and 770 nm from composite α/β–Bi_2_O_3_ attributed to defect/impurity states induced by oxygen vacancies present in the nanostructures [47]. Recently, a schematic study carried out by Schmidt et al. reported an enhanced red-band emission from a sample with a high density of V_O_ in α–Bi_2_O_3_ [51]. Wu et al. affirmed that the higher PL emission intensity of red-band emission means a higher V_O_ density. Furthermore, luminescence is strongly affected by the process of sample preparation [52]. Therefore, under UV excitation, the red-band emission ~701 nm arises when the photogenerated holes trapped in the deep-level V_O_ recombine with the electrons trapped at a shallow level located just below the conduction band.

**Figure 5:**
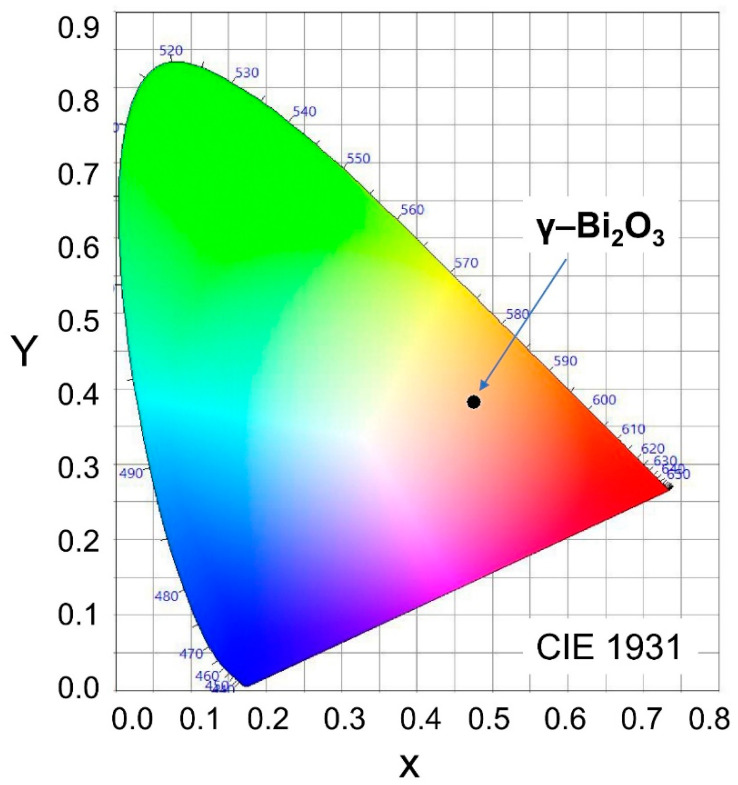
CIE 1931 color space chromaticity diagram in the (*x*, *y*) coordinates system showing orange color (black dot) of the PL emission from γ–Bi_2_O_3_ NPs.

The international commission on illumination (CIE) 1931 color space chromaticity diagram in the (x, y) coordinates system shows the orange color of the PL emission from γ–Bi_2_O_3_ NPs (Figure 5). The chromaticity coordinates (0.4759, 0.3819) with correlated color temperature (CCT) is 2274 K for γ–Bi_2_O_3_ NPs.

## 4. Discussion

Now we ask, what could be the possible mechanism behind the RT stabilization of γ–Bi_2_O_3_? Based on the above analysis, it appears that the excess V_O_ generated within γ–Bi_2_O_3_ may have played an important role in RT stabilization [53]. The excess V_O_ could be residing either at tetrahedral O(3) sites [16] or at the octahedral O(1) and/or O(2) sites. As discussed in the Introduction, there exists a high density of intrinsic V_O_ defects on the surface of γ–Bi_2_O_3_ tetrahedra, i.e., O(3) (8c) sites [15]. However, according to theoretical calculations, the introduction of V_O_ at O(3) sites makes the crystal structure of γ–Bi_2_O_3_ further distorted, due to which it loses its I23 symmetry. Therefore, as observed from PXRD analysis, it is possible that during the annealing process, a high density of V_O_ defects could have been formed in γ–Bi_2_O_3_ at octahedra O(2) sites (in the internal Bi–O framework) such that the excess defects may have prevented the transformation from BCC to monoclinic α–Bi_2_O_3_ phase, which, in turn, resulted in the formation of RT stabilized γ–Bi_2_O_3_ NPs [26]. In conclusion, RT stabilized γ–Bi_2_O_3_ NPs with a mean diameter of 90 nm were successfully prepared at the expense of Bi NPs simply by annealing at 550 °C for a duration of 2 h in an ambient atmosphere. The proposed method is very mild, which eases the mass production of γ–Bi_2_O_3_ NPs. PXRD and Raman spectroscopy confirmed the RT stabilization of formed single-crystalline BCC γ–Bi_2_O_3_ NPs. The observed red-shift and broadening in the phonon modes associated with the inhomogeneously distorted Bi–O lattice were ascribed to Bi and oxygen vacancy defects. γ–Bi_2_O_3_ NPs exhibited a strong red-band emission, peaking at ~701 nm, and covering 81% integrated intensity of the PL spectra. Our findings suggest that the RT stabilization and enhanced red-band emission in γ‒Bi_2_O_3_ is mediated by excess oxygen ion vacancies generated at the octahedra O(2) sites during the thermal annealing process in an ambient atmosphere, as observed from PXRD. The new finding in this study is valuable in terms of providing a fundamental understanding of the RT stabilization of γ–Bi_2_O_3_ for future use as a photocatalyst.

## Figures and Tables

**Table 1:** Rietveld refined parameters for XPRD spectra of γ–Bi_2_O_3_ NPs. All structural and lattice parameters were allowed to very simultaneously until the weighted wR factor and the goodness of fitting (GOF), differed by less than one part in a thousand in two successive cycles ^1^.

Sites	Element	*x*	*y*	*z*	Occupancy
24f	Bi(1)	0.8244(1)	0.6801(1)	0.9881(1)	0.9989(4)
2a	Bi(2)	0	0	0	0.943(8)
24f	O(1)	0.8690(7)	0.7661(7)	0.4884(16)	1.0104(17)
8c	O(2)	0.8093(16)	0.8093(16)	0.8093(16)	0.861(29)
8c	O(3)	0.162(3)	0.162(3)	0.162(3)	1.08(4)

^1^ lattice parameter *a* = *b = c* =10.1115(1) Å; volume *V* = 1033.82(3) Å^3^; Space group I23 (No. 197); wR(%) = 2.460; goodness of fitting (GOF) (%) = 4.71.

**Table 2:** Summary of deconvoluted Raman spectra of γ–Bi_2_O_3_ NPs.

Vibration Symmetry [41]	Frequency (cm^−1^)	FWHM (cm^−1^)	I.A. (%)
A	81	10	3.2
A	89	15	3.0
A	141	14	3.0
A	157	23	2.9
A	281	28	6.6
A	312	36	14.6
A	528	34	24.0
E/F	111	23	5.6
E	125	10	4.9
E	257	27	9.3
E	381	30	4.0
E	448	11	0.2
E	614	16	0.2
F	206	14	0.3
F	344	54	13.2
F	436	12	0.05
F	494	26	1.6
F	628	24	1.1
?	567	22	2.3
?	481	5	0.05

**Table 3:** Summary of deconvoluted photoluminescence spectra of γ–Bi_2_O_3_ NPs.

Peak Center (nm)	FWHM (nm)	Integrated Area (a.u.)
394	57	259
487	115	912
588	61	1657
701	120	11,936

## Supplementary Materials

The following are available online at https://www.mdpi.com/2079-4991/10/6/1023/s1, Figure S1: Total 9 SEM images and EDS spectra with points, from which the chemical data is collected, Table S1: Summary of Bi and O atomic percentage collected from EDS spectra and their average value.
