# Supplementary material for: Structural and Enhanced Optical Properties of Stabilized γ‒Bi2O3 Nanoparticles: Effect of Oxygen Ion Vacancies
Source: Nanomaterials (Basel). 2020 May 27;10(6):1023. doi: 10.3390/nano10061023 (PMC7352962; doi:10.3390/nano10061023)
Supplement: Supplementary file 1 [file nanomaterials-10-01023-s001.pdf]

Appendix A: Supporting Information

Structural and enhanced optical properties of stabilized γ–Bi_2O_3 nanoparticles: Effect of oxygen ion vacancies

Ashish Chhaganlal Gandhi, Chia-Liang Cheng, and Sheng Yun Wu*

Department of Physics, National Dong Hwa University, Hualien 97401, Taiwan

Figure S1 Total 9 SEM images and EDS spectra with points, from which the chemical data collected is as shown below:

A

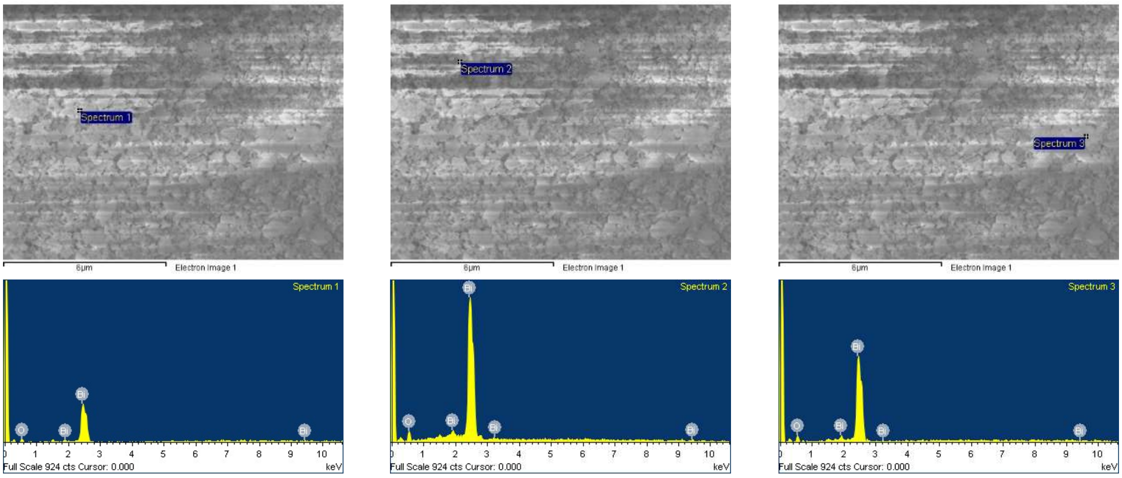

B

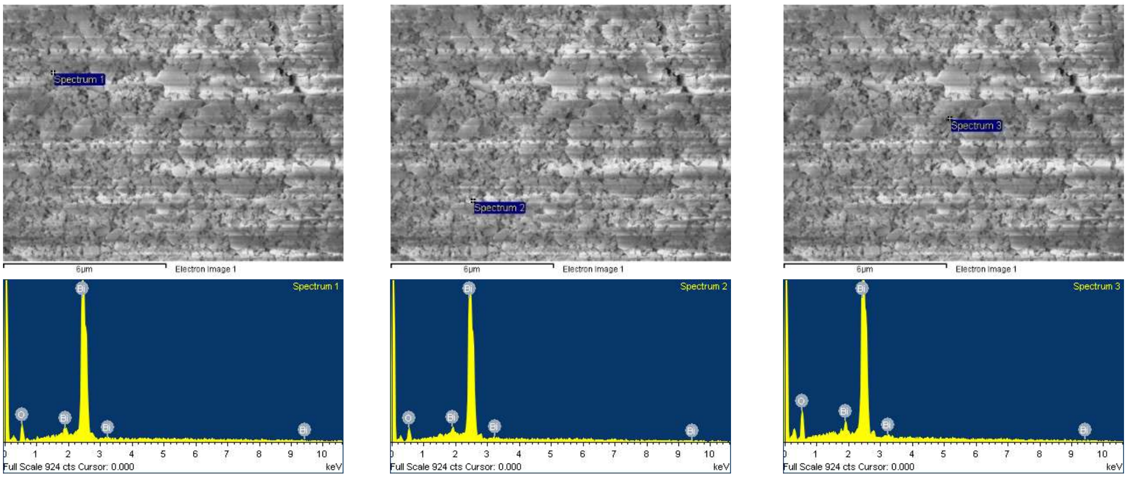

C

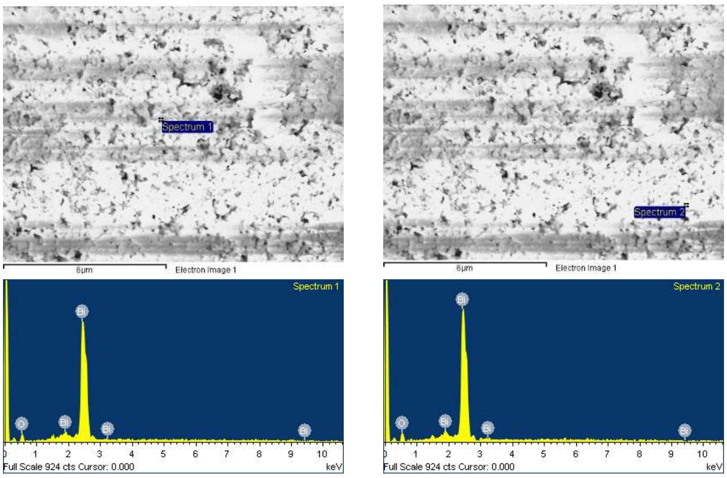

D

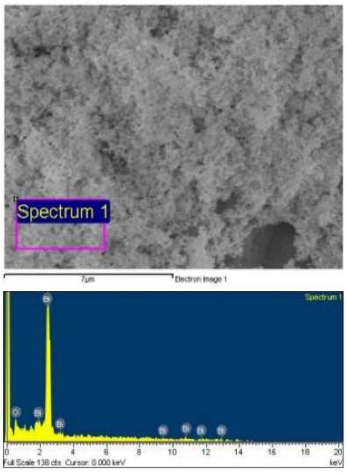

Table S1 Summary of Bi and O atomic percentage collected from EDS spectra and their average value.

No.	Spectrum	Bi (atomic %)	O (atomic %)
1	A-Spectrum 1	47.48	52.52
2	A-Spectrum 2	49.47	50.53
3	A-Spectrum 3	48.64	51.36
4	B-Spectrum 1	42.97	57.03
5	B-Spectrum 2	47.43	52.57
6	B-Spectrum 3	32.81	67.19
7	C-Spectrum 1	54.57	45.43
8	C-Spectrum 2	51.18	48.82
9	D-Spectrum 1	43.97	56.03
**Average**		**46.50**	**53.50**
